# Mosquito host background impacts microbiome-Zika virus interactions in field- and laboratory-reared *Aedes aegypti*

**DOI:** 10.1186/s42523-025-00482-0

**Published:** 2025-11-05

**Authors:** Cintia Cansado-Utrilla, Miguel A. Saldaña, George Golovko, Kamil Khanipov, Riley K. Watson, Alexander L. Wild, Laura E. Brettell, Scott C. Weaver, Eva Heinz, Grant L. Hughes

**Affiliations:** 1 Departments of Vector Biology and Tropical Disease Biology, Liverpool School of Tropical Medicine, Liverpool, UK; 2 Mosquito and Vector Control Division, Harris County Public Health, Houston, TX USA; 3 Department of Microbiology and Immunology, Institute for Human Infections and Immunity, University of Texas Medical Branch, Galveston, TX USA; 4 Department of Pharmacology and Toxicology, The University of Texas Medical Branch, Galveston, TX USA; 5 Department of Integrative Biology, University of Texas, Austin, TX USA; 6 School of Science, Engineering and Environment, University of Salford, Manchester, M5 4WT UK; 7 Departments of Vector Biology and Clinical Sciences, Liverpool School of Tropical Medicine, Liverpool, UK; 8 Department of Microbiology and Industrial Biotechnology, Institute of Pharmacy & Biomedical Sciences, University of Strathclyde, Glasgow, UK

## Abstract

Mosquito microbiota abundance and composition are modulated by a variety of factors, including pathogen exposure. The microbiome can also influence pathogen infection of the host and thus harbours considerable potential to impact transmission of pathogens. As such, there is a growing interest in using particular bacterial members of the microbiota for novel vector-control strategies. However, before novel microbiota-based approaches can move towards translation, a more complete understanding of the interactions between mosquitoes, their microbiome, and the pathogens they transmit, is required to better appreciate how variation in the microbiome of field mosquitoes affects these interactions. To examine the impact of the mosquito background and the associated diversity of bacterial microbiota within distinct hosts, we exposed several laboratory-reared and field-collected *Aedes aegypti* mosquito lines to Zika virus (ZIKV) and examined their bacterial load and composition in response to pathogen exposure and viral infection success. Intriguingly, we show that ZIKV exposure and infection had distinct impacts on microbiome composition and density within different mosquito lines. In one laboratory-reared line ZIKV exposure and infection reduced the bacterial load, while conversely in another line load was increased by the virus. Distinct responses of the microbiome were also seen in mosquitoes collected from the field. Sampling site-specific differences in the microbiome of mosquitoes were observed as virus infection altered microbiome alpha and beta diversities in one cohort, while in mosquitoes from other sampling sites, viral progression through the mosquito had minimal effect on the microbiome. We also identified bacterial taxa correlating with either ZIKV infection or a lack of infection. These taxa are potential candidates for future follow-up studies disentangling functional mechanisms and directionality of interactions. Overall, our study highlights that interactions between mosquito, virus, and microbiota are variable and context dependent, and that tripartite interactions among distinct mosquito cohorts and their microbiomes are not universal.

**Supplementary Information:**

The online version contains supplementary material available at 10.1186/s42523-025-00482-0.

## Author summary

The mosquito microbiome composition can vary greatly between individuals and populations. Microbiota profoundly affect pathogen transmission in mosquitoes, while conversely, infection with a pathogen can alter the mosquito microbiome. However, we have a poor understanding of how universally conserved these pathogen-related effects are between mosquito populations. To address this, we infected different lab-reared mosquito lines and examined their microbiomes after exposure to Zika virus (ZIKV) compared to unchallenged mosquitoes. Importantly, we also conducted experiments with field-caught mosquitoes which have markedly different microbiomes compared to their lab-reared counterparts. Here we gave all mosquitoes virus-infected blood meals and compared the microbiomes of individuals that became infected to those that did not. The observed microbiome changes differed between host lines. This could be due to different baseline microbiota in their respective host lines, or differences in the hosts’ response to viral infection which subsequently alters the microbiome in a distinct manner. As members of the microbiota are being evaluated for novel approaches to control mosquito-borne disease, our findings are highly relevant to developing a more complete understanding of host-microbe-pathogen interactions. Variation of the microbiome among different mosquito lines needs to be considered in experimental designs, in the interpretation of infection results, and for the deployment of interventions in the field.

## Background

The mosquito and its associated microbial community collectively form the mosquito holobiont, a complex ecosystem with multi-layered interactions [1]. Host-microbe interactions influence several phenotypes of mosquitoes such as growth and development, reproduction, and the ability to transmit pathogens, all of which are important for vectorial capacity [2]. Microbiome composition is influenced by the host background but also by multiple other factors including environmental parameters, microbe-microbe interactions and exposure to pathogens [3–10]. Different microbiota composition and abundance could explain the variation seen in vector competence of different mosquito lines to viruses [11–15].

Interactions between bacterial microbes and pathogens are bi-directional with the microbiome affecting the outcomes of infection with human pathogens [16–22], and conversely pathogen infection altering the microbiome composition and abundance [17, 23–25]. Bi-directional interactions can be mediated indirectly by host immunity, given that both pathogens and bacteria elicit and are modulated by these pathways [26, 27]. Additionally, microbiota can directly affect pathogen infection by secreted compounds and metabolites that influence parasites or arboviruses [28–30]. These direct microbiota-pathogen interactions can either positively or negatively affect mosquito susceptibility to pathogens. For instance, in *Aedes aegypti,* some isolates of *Serratia* have been implicated in enhancing susceptibility to dengue virus (DENV) infection, whereas members of the *Rosenbergiella* genus impair vector competence to both DENV and Zika virus (ZIKV) [28, 30]. These and other studies have provided robust evidence that specific bacterial taxa influence vector competence, however we were interested in understanding how the microbiome collectively interacts with arboviruses and vice versa, and how conserved these observed interactions are between different mosquito host backgrounds.

Much of our insight into the tripartite interactions between the host, their bacterial microbiota, and pathogens is derived from laboratory-based studies on inbred mosquito lines, where the involvement of the microbiome is often assessed by perturbation. This is typically achieved by administration of antibiotics to alter the microbiome; either a single compound [20] or a cocktail [4, 31]. Using especially broad-spectrum treatments can also impact host fitness and mitochondria [21]. Antibiotic treatments do not necessarily completely eliminate microbiota, but rather generates a highly artificial situation of a limited or a heavily biased microbiome [4, 31, 32]. Alternatively, bacteria can be introduced into mosquitoes either at the aquatic stages in the larval water, or to adults via a sugar meal, thereby seeding specific taxa into an already established microbiome. This may reduce the level of disruption of the holobiont system, and mimic administration approaches that could occur in control interventions. While such manipulation experiments provide evidence for the microbiome’s role in vector competence and provide candidates for microbial control, they do not comprehensively address how variability in an established microbiome influences tripartite interactions.

In this study, we investigated how natural microbiome variability influences the interactions between distinct *Ae. aegypti* mosquito lines, their microbiota, and ZIKV. To address how differences in the microbiota between and within mosquito populations altered interactions with ZIKV, we collected host-seeking females from different geographic regions in Texas (USA), provided them with an infectious ZIKV blood meal, and monitored viral infection status, viral loads post infection, and bacterial microbiome composition. Additionally, using two different laboratory-reared *Ae. aegypti* colonies also originating from Texas, we examined if microbiota responded to pathogen infection in a similar fashion in differing host backgrounds. Our results highlight the complexity of tripartite interactions in mosquitoes, and how variation in the host and its microbiome dictate these interactions.

## Methods

### Mosquito lines and collections

Field mosquitoes were collected outdoors over a three-day period, in Austin (30°19’38.4“N 97°45’06.5“W), Galveston (29°18’23.1“N 94°46’51.2“W), and Brownsville (25°52’44.3“N 97°26’19.2“W), Texas, USA. On each day, host-seeking mosquitoes were captured using CDC Fay-Prince traps for three hours at dawn and dusk, with collection cups replaced every hour. Mosquitoes were retrieved from traps and stored in large cartons kept within plastic bins containing a moist sponge for humidity and provided with 10% sucrose until their arrival at the insectaries of the University of Texas Medical Branch (UTMB) (Galveston, Texas, USA). Mosquitoes were then anesthetized at 4 °C and their species and sex were determined by morphological identification. Female *Ae. aegypti* were transferred to new cartons, provided 10% sucrose, and stored in incubators (27 °C and 80% humidity) within a secondary plastic bin containing a moist sponge to increase humidity. Laboratory reared mosquito lines used in this study were Galveston and Rio Grande Valley (RGV), two recently established colonies at UTMB, the former for three generations and the latter for six. All mosquito lines were maintained under standard insectary conditions at UTMB (27 °C and 80% humidity). Larvae were fed with fish food while adults were fed with 10% sucrose that was autoclaved prior to use.

### Viral strains and mosquito infections

**Fig. 1:**
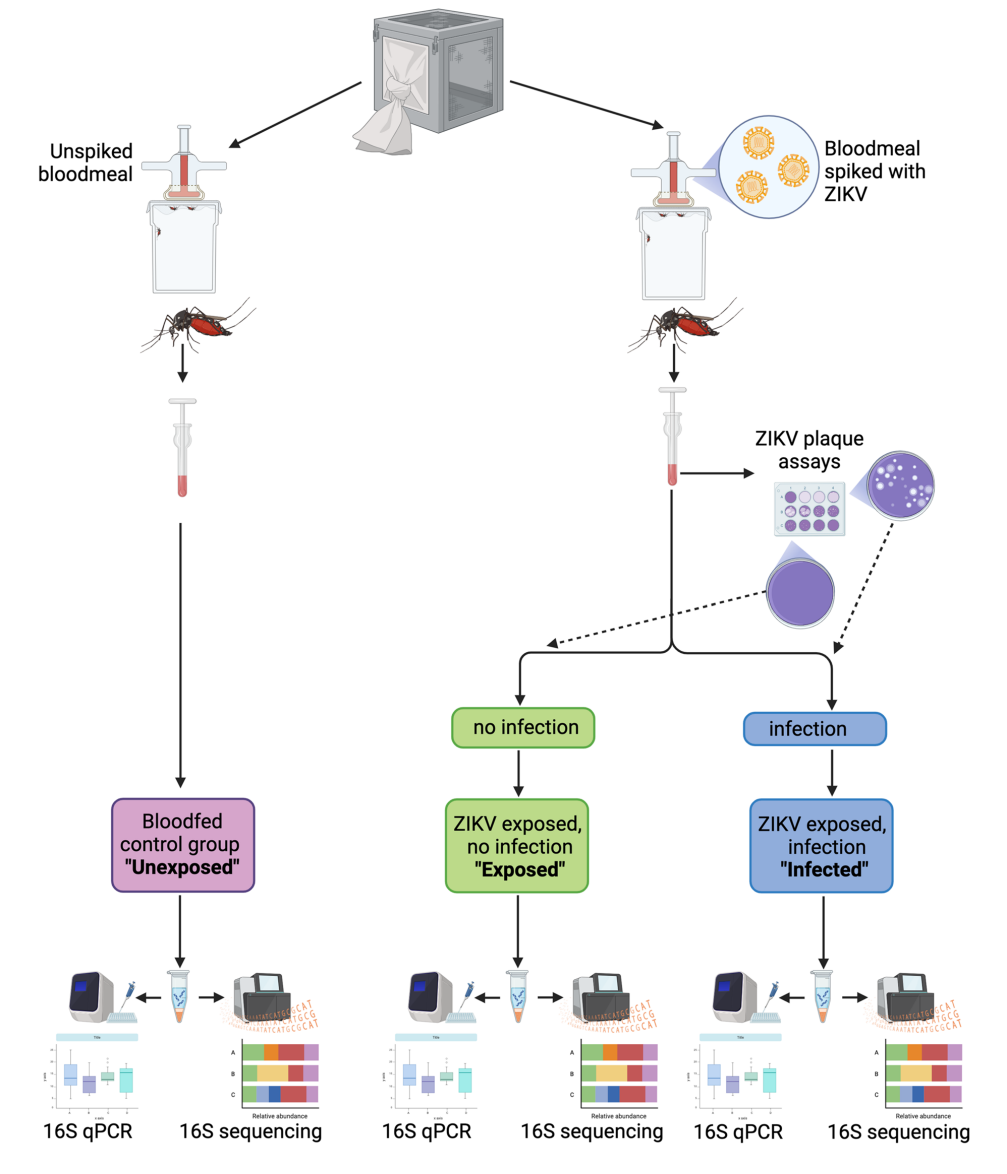
Experimental design for ZIKV infection of lab-reared *Ae. aegypti* lines. After ZIKV infectious blood meals mosquitoes were designated into groups termed “exposed” indicating exposure but a lack of infection, or “infected”, indicating infection of ZIKV in mosquitoes. An “unexposed” group consisted of blood meal without virus. Figure created using biorender.com

The viral strain used in this study was ZIKV MEX 1–7 (KX247632.1), isolated from *Ae. aegypti* in Mexico in 2016 [33]. The virus was acquired as a lyophilized stock from the World Reference Center for Emerging Viruses and Arboviruses at UTMB. It was cultured in C6/36 cells, an *Ae. albopictus*-derived cell line, followed by four passages in the mammalian Vero cell line to generate stocks. Vero cells were maintained in high-glucose Dulbecco’s modified Eagle’s medium (DMEM) supplemented with 5% foetal bovine serum (FBS) and 1% penicillin/streptomycin at 37 °C and 5% CO_2_. Laboratory-reared and field-collected mosquitoes were starved for 18 hours before being offered a blood meal spiked with ZIKV (10^6^ FFU/ml) (Austin *N* = 113, Galveston *N* = 40, Brownsville *N* = 19, Galveston-lab *N* = 57, RGV-lab *N* = 85). Bloodmeals were offered five days post-pupal eclosion to lab mosquitoes and one to three days post collection to field mosquitoes. Mosquitoes that did not feed were removed. Galveston and RGV lab-reared mosquitoes were offered an uninfected bloodmeal (Galveston-lab *N* = 40, RGV-lab *N* = 40) as a control. Ten days after blood feeding, mosquitoes were euthanised and those that had been provided with an infected bloodmeal were assessed for ZIKV infection using focus forming assays. The microbiomes of all mosquitoes were characterised using qPCR and 16S rRNA amplicon sequencing (Fig. 1).

### Focus forming assay

Individual mosquitoes that had fed on an infected bloodmeal were surface sterilized (5 minutes in 70% ethanol followed by three washes in PBS for five minutes each) and homogenized in 500 µl of tissue culture medium (DMEM supplemented with 5% FBS, 1% penicillin/streptomycin and 1% amphotericin) using a TissueLyser II (Qiagen) for five minutes at 60 Hz. Mosquito samples were serially diluted and inoculated onto Vero cells in 48-well plates and overlaid with 0.8% methylcellulose in DMEM. Mosquito bodies and legs were used to determine viral infection or dissemination, respectively. Plates were washed with PBS, incubated at 37 °C for four days and fixed with 50:50 methanol:acetone. Foci were stained using a mouse hyperimmune polyclonal anti-ZIKV primary antibody (World Reference Center for Emerging Viruses and Arboviruses, UTMB) and HRP-labelled goat anti-mouse secondary antibody (KPL, Gaithersburg, MD). ZIKV foci were then visualized using an aminoethylcarbazole (AEC) detection kit (Enzo Diagnostics, Farmingdale, NY) according to the manufacturer’s protocol.

### Estimation of bacterial density

Genomic DNA was extracted from 250 µl of the homogenate, obtained from the material used for focus forming assay, using the NucleoSpin Tissue kit (Macherey-Nagel) as previously described and used as template for qPCR [34]. Universal bacterial 16S ribosomal RNA primers and the housekeeping S7 gene primers were used as previously described [34–36]. Relative gene expression was calculated using the 2^-ΔΔCt^ method [37]. Microbiome load (16S/S7) data were analysed in RStudio (version 1.4.1717), density and Q-Q plots with the *ggpubr* package (version 0.6.0) and Shapiro-Wilk tests using the *stats* package (version 4.3.2) [38, 39]. The data was not normally distributed in any of the groups, so Wilcoxon-Rank Test was used to compare the means using the *ggpubr* package (version 0.6.0).

### Analysis of 16S rRNA amplicon sequences

Genomic DNA from all mosquitoes was then used for high-throughput sequencing targeting the bacterial 16S rRNA gene. Sequencing libraries for each isolate were generated using universal 16S rRNA V3–V4 region primers following Illumina 16S rRNA metagenomic sequencing library protocols [40]. The samples were barcoded for multiplexing using Nextera XT Index Kit v2. Sequencing was performed on an Illumina MiSeq instrument using a MiSeq Reagent Kit v2 (500 cycles). Quality control and taxonomical assignment of the resulting reads was performed using CLC Genomics Workbench 8.0.1 Microbial Genomics Module (http://www.clcbio.com). Low quality reads containing nucleotides with a quality threshold below 0.05 (using the modified Richard Mott algorithm), as well as reads with two or more unknown nucleotides or sequencing adapters were removed. Reference based OTU selection was performed using the SILVA SSU v128 97% database [41]. Sequencing of 16S failed for seven samples (five field collected individuals (Austin) and two unexposed individuals (RGV)). Chimeras were removed from the dataset if the absolute crossover cost was 3 using a k-mer size of 6. Data were then transferred to RStudio for subsequent analyses. Samples with fewer than 2,000 reads were removed (18 from Austin, one from Galveston-field, one from Brownsville, two from Galveston-lab and six from RGV-lab), resulting in a final data set comprising 359 samples (90 from Austin, 39 from Galveston-field, 18 from Brownsville, 95 from Galveston-lab and 117 from RGV-lab; (Table S1; Figure S1)). Data were then converted to a phyloseq object using the *Phyloseq* package (version 1.46.0) [42]. To assess whether contaminants had been introduced to the samples during processing or sequencing, we applied a filtering threshold to the dataset, removing any OTU present at < 10 reads or < 0.1% of the total dataset [3]. This resulted in 1384/1406 OTUs being removed from the dataset, many of which were commonly observed mosquito symbionts (eg *Asaia*, *Serratia*, *Pantoea*) (Table S2) [1, 2, 15]. As such, we concluded that this filtering would likely result in the removal of true symbionts, so continued analysis with the full dataset without the removal of reads. Diversity parameters (Shannon entropy and Bray-Curtis distance) were assessed using the *vegan* package (version 2.6–4) [43]. Shannon diversity index data were tested for normality using density and Q-Q plots and Shapiro-Wilk tests. All data groups failed tests for normality, so a Wilcoxon-Rank Test was used to compare the means. Overall differences in beta diversity between groups was carried out using permutational multivariate analysis of variance (PERMANOVA) testing using the ‘Adonis2’ function in the *vegan* package with subsequent pairwise testing using the *PairwiseAdonis* package (version 0.4.1) [44]. Beta diversity was visualised using NMDS plots and ellipses were added to the plots using the ‘stat_ellipse’ function in *ggplot2* using the default 95% confidence levels assuming multivariate t-distribution [45]. Determination of differentially abundant taxa between groups was calculated at the genus level using Analysis of compositions of microbiomes with bias correction (ANCOM-BC, version 2.5.0) [46]. Differentially abundant taxa between RGV-lab mosquitoes and Galveston-lab mosquitoes were identified separately for the unexposed, exposed and infected groups. Pairwise tests between lines were conducted using a log linear model fitted with the formula ~ZIKV, where ZIKV corresponds to infection status and default parameters, with the exception of using the ‘bonferoni’ p-value adjustment method. A heatmap was then generated to show the relative abundance of the differentially abundant taxa (adjusted *p* value < 0.05) in each group between Galveston and RGV mosquitoes using the *pheatmap* package [47]. When comparing between the unexposed, exposed and infected groups in each of the RGV-lab and Galveston-lab mosquitoes, the ‘ancombc2’ function was used to perform multiple pairwise comparisons. Taxa with an adjusted *p* value of < 0.05 and passing the sensitivity analysis were classed as differentially abundant.

## Results

### Mosquito line influences the ZIKV-microbiome interaction

**Fig. 2:**
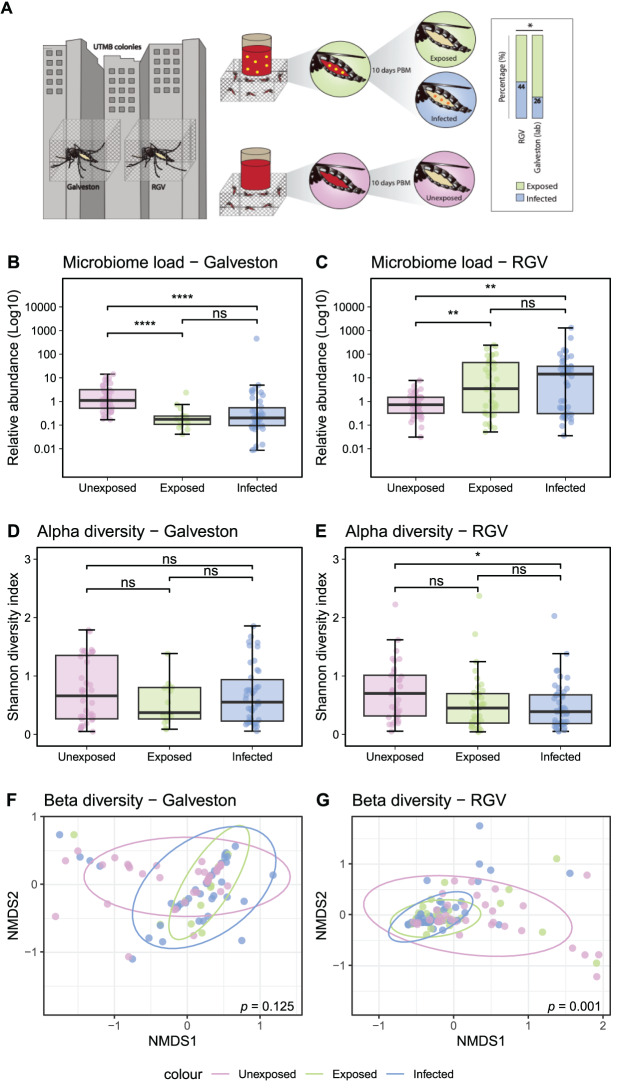
Viral infection of lab-reared mosquitoes and impact on the microbiome. Two *Ae. aegypti* lines, Galveston and Rio Grande Valley (RGV) were reared in the insectaries of UTMB. One cohort from each line was offered a bloodmeal (red) spiked with ZIKV (yellow) (Galveston *n* = 57, RVG *n* = 85). A second cohort from each line (*n* = 40 mosquitoes per line) were offered an uninfected bloodmeal (unexposed, pink). Ten days post bloodmeal (PBM) infection was assessed and mosquitoes were classified in exposed (ZIKV was not detected) (green) or infected (ZIKV was detected) (blue). Infection rate was assessed (right) and statistical difference is shown as * (chi-square, *p* < 0.05) (**A**). Relative abundance of bacterial 16S rRNA was measured in Galveston (**B**) and RGV (**C**) mosquitoes. Alpha diversity (Shannon diversity index) of the microbiome was assessed in Galveston (**D**) and RGV (**E**) mosquitoes. Statistical differences are shown as **** (*p* < 0.0001), ** (*p* < 0.01), * (*p* < 0.05) and ns (non-significant) (wilcoxon rank test). Beta diversity of the microbiome was assessed in Galveston (**F**) and RGV (**G**) mosquitoes. *p* values show results of PERMANOVA analysis of bray-curtis dissimilarity. Subsequent pairwise testing of beta diversity in the RGV group showed there were statistically significant differences between both unexposed vs. exposed and unexposed vs. infected mosquitoes (both *p* < 0.003)

To investigate whether interactions between ZIKV and the microbiome differ between different *Ae. aegypti* backgrounds, we fed two laboratory-reared *Ae. aegypti* lines (Galveston-lab and RGV-lab) with either a non-infectious bloodmeal (unexposed control group) or a blood meal spiked with ZIKV. Subsequently, we assessed the latter group for viral infection and categorised them as exposed (no ZIKV infection detected) or infected (ZIKV infection detected). Proportion of infected individuals differed significantly between lines, with 44% infection in RGV-lab mosquitoes and 26% infection in Galveston-lab mosquitoes (Chi-square = 4.87, df = 1, *p* = 0.03) (Fig. 2A).

To assess whether ZIKV affected the microbiomes of these two distinct laboratory-reared mosquito lines in a similar fashion, we compared density, diversity, and composition of the microbiome among the three groups (unexposed, exposed, and infected) for each host line. In the Galveston-lab line, ZIKV exposure and infection led to a reduction in bacterial density compared to unexposed (Wilcoxon Rank test W = 539, *p* < 0.001 exposed vs. unexposed, and W = 1343, *p* < 0.001 infected vs. unexposed) (Fig. 2B). Conversely, in the RGV-lab line, ZIKV exposure and infection resulted in an increase in bacterial density (Wilcoxon Rank test W = 485, *p* = 0.009 exposed vs. unexposed, and W = 575, *p* = 0.001 infected vs. unexposed) (Fig. 2C). In the Galveston-lab line, neither ZIKV exposure nor infection caused significant differences in alpha or beta diversity (Fig. 2D, F). However, ZIKV infection led to a significant reduction in Shannon diversity of the RGV-lab line microbiome (Wilcoxon Rank Test, W = 1029, *p* = 0.013) (Fig. 2E), while both exposure and infection significantly altered beta diversity compared to unexposed (pairwise adonis test, *p* = 0.003 exposed vs. unexposed and *p* = 0,003 infected vs. exposed) (Fig. 2G).

**Fig. 3:**
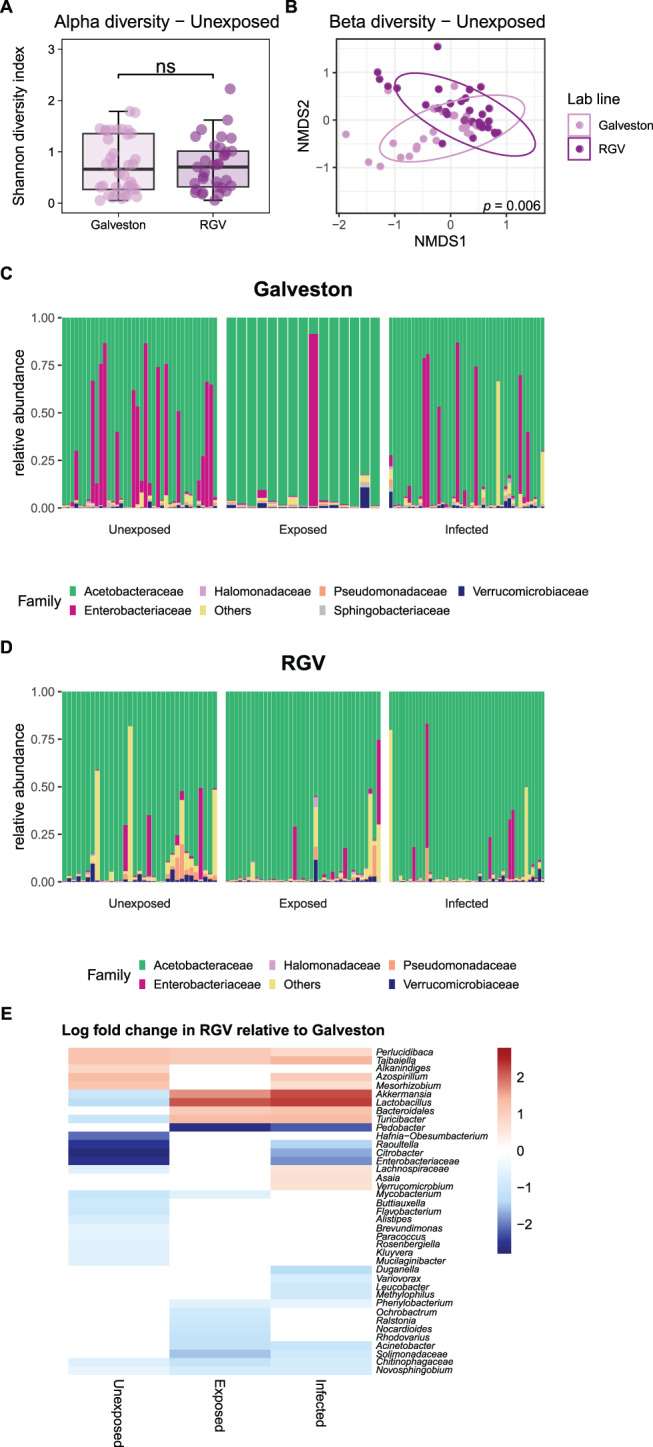
Comparison of microbiome diversity between *Ae. aegypti* laboratory lines. Alpha diversity (**A**) and beta diversity (**B**) were assessed in unexposed RGV and Galveston mosquitoes. Statistical differences are shown as ns (non-significant) (wilcoxon rank test). *p* value shows results of PERMANOVA analysis of bray-curtis dissimilarity. Relative abundance of bacterial families was explored in Galveston (**C**) and RGV (**D**) mosquitoes either unexposed, ZIKV exposed or ZIKV infected. The heatmap shows the ANCOM-BC results (adjusted *p*-value < 0.05) of enriched taxa (red) or depleted taxa (blue) in RGV mosquitoes in comparison with Galveston mosquitoes within the unexposed, ZIKV-infected and ZIKV-exposed groups (**E**)

To evaluate whether the native microbiome was different between the two mosquito lines, we examined the diversity of the unaltered (ZIKV-unexposed) microbiome. While no significant difference was observed in alpha diversity between the lines (Fig. 3A), beta diversity displayed a significant difference (PERMANOVA, *p* = 0.006) (Fig. 3B). In the two distinct lab lines, analysis of microbiomes of unexposed, exposed and infected showed that irrespective of ZIKV infection status, both host lines were dominated by *Acetobacteraceae* (Fig. 3C, D), while members of the *Enterobacteriaceae* family were notable in the Galveston-lab line.

Testing for differential abundance in the microbiome composition between the Galveston-lab and RGV-lab lines, considering each condition, showed that a total of 39 taxa exhibited significant differential abundance between the two lines when comparing each condition separately (Fig. 3E), although the majority of these taxa were at low prevalence across the dataset (Table S2). *Turicibacter*, *Akkermansia* and *Lactobacillus* showed the most pronounced changes. These bacteria had higher relative abundances in Galveston-lab mosquitoes in the unexposed cohort but this shifted in the infected and exposed groups with increases in the RGV-lab line. Conversely, both ZIKV exposure and infection resulted in a relative decrease of *Pedobacter* in RGV-lab mosquitoes compared to Galveston-lab mosquitoes.

### Bacterial taxa correlate with ZIKV infection in Ae. aegypti

**Fig. 4:**
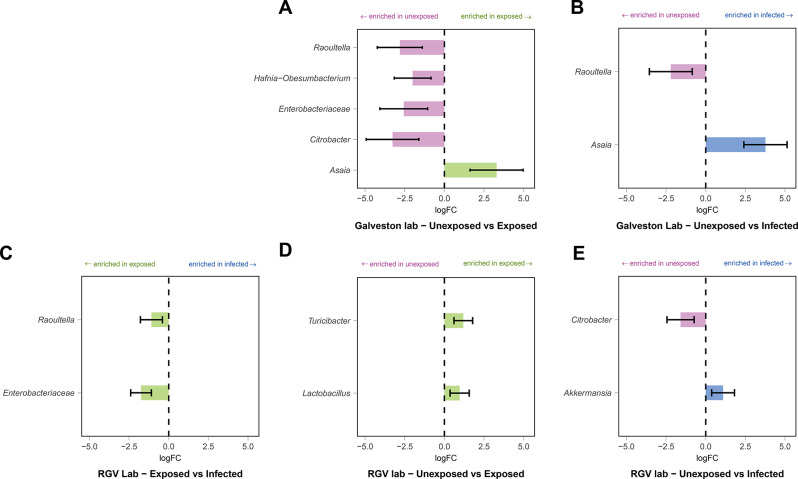
Differential abundance of microbes based on infection status. ANCOM-BC2 was used to identify taxa that were differentially abundant in pairwise comparisons between unexposed, exposed and infected mosquitoes. Only taxa classified as significant based on an adjusted *p*-value < 0.05 and passing the sensitivity analysis are shown. Comparisons are shown for Galveston (**A, B**) and RGV (**C-E**) mosquitoes, comparing unexposed to exposed (**A, D**), unexposed to infected (**B, E**) and exposed to infected (**C**). Colours indicate taxa enriched in unexposed (pink), exposed (green) and infected (blue) mosquitoes. No differentially abundant taxa were identified between exposed and infected Galveston mosquitoes

Next, we examined the differential abundance of microbiome members, comparing the infection status (unexposed, exposed and infected) in both the RGV-lab and Galveston-lab lines. We found differentially abundant bacteria in Galveston-lab mosquitoes when comparing unexposed to exposed (Fig. 4A), and unexposed to infected (Fig. 4B), while differentially abundant bacteria were seen in all three pairwise comparisons in the RGV-lab mosquitoes (Fig. 4C–E). *Asaia* was significantly enriched in exposed and infected compared to unexposed Galveston-lab mosquitoes, while *Raoultella* was depleted (Fig. 4A–B). Conversely, *Raoultella* was enriched in exposed relative to infected RGV-lab mosquitoes along with Enterobacteriaceae. *Citrobacter* was enriched in unexposed RGV-lab mosquitoes when compared to infected whereas this genus was significantly enriched in the unexposed Galveston-lab mosquitoes when compared to the exposed group. Many of the bacteria identified as differentially abundant between groups, including *Asaia*, *Raoultella* and *Citrobacter* are dominant taxa across the dataset (Table S2).

### Microbiome-ZIKV interactions in field-collected mosquitoes

**Fig. 5:**
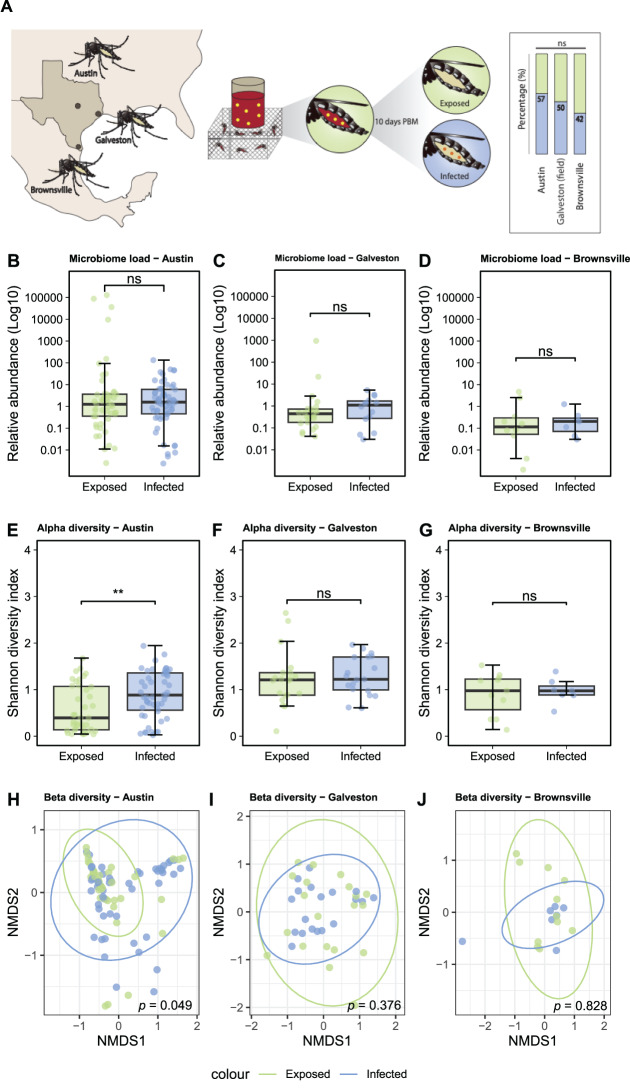
ZIKV infection of field-collected *Ae. aegypti* mosquitoes and impact of virus on the microbiome load and diversity. Field collected *Ae. aegypti* mosquitoes were collected from three locations in Texas; Austin (*N* = 113), Galveston (*N* = 40) and Brownsville (*N* = 19), and offered a ZIKV infected blood meal. infection was assessed and mosquitoes were classified in exposed (ZIKV was not detected, green) or infected (ZIKV was detected, blue). Infection rate was assessed (right) and statistical difference is shown as * (chi-square, *p* < 0.05) (**A**). Relative abundance of bacterial 16S rRNA in Austin (**B**), Galveston (**C**) and Brownsville (**D**) mosquitoes. Alpha diversity (Shannon diversity index) of the microbiome in Austin (**E**), Galveston (**F**) and Brownsville (**G**) mosquitoes. Statistical differences are shown as ** (*p* < 0.01) and ns (non-significant) (Wilcoxon rank test). Beta diversity of the microbiome in Austin (**H**), Galveston (**I**) and Brownsville (**J**) mosquitoes. Pairwise PERMANOVA was used for statistical analysis of the Bray-Curtis dissimilarity distance of microbiomes (bottom right of panel)

In field-collected mosquitoes the prevalence of infection following exposure was comparable across sites, with infection rates recorded at 57%, 50% and 42% in mosquitoes collected in Austin, Galveston, and Brownsville, respectively (Fig. 5A). Comparing Galveston-field to Galveston-lab mosquitoes exposed or infected groups showed that both the alpha (reduced in lab-reared mosquitoes compared to field-caught) and beta diversity were significantly different (Figure S2). When we examined the relative abundance of bacterial taxa in exposed and infected mosquitoes, no taxa showed significant differential abundance when comparing infected and exposed groups. *Acetobacteraceae* represented the major microbiome component in Austin-field mosquitoes, while *Pseudomonadaceae* were more prevalent in Galveston-field mosquitoes (Figure S3).

A comparative analysis of the microbiome between exposed and infected mosquitoes from each field site showed no differences in the bacterial load following viral infection in mosquitoes from any location (Fig. 5B–D). However, when examining the diversity of the microbiome in exposed and infected mosquitoes from each location, significant differences in alpha (Wilcoxon Rank Test, *p* < 0.01) and beta (PERMANOVA, *p* = 0.049) diversity was uniquely observed in mosquitoes collected from Austin (Fig. 5E–J).

## Discussion

While a range of diverse arboviruses have been shown to alter the mosquito microbiome [8–10, 17, 25], we have a poor understanding regarding how universal these phenotypes are in divergent mosquito lines. Using laboratory-reared mosquitoes we showed that the density of microbiota in different mosquito lines responded distinctly, with bacterial load increasing in the RGV-lab line yet decreasing in the Galveston-lab line in response to ZIKV exposure and infection. Importantly, we also found variable effects of viral infection and exposure on the microbiome in field-collected mosquitoes. Infection altered both alpha and beta diversity of mosquito microbiomes collected in Austin, but not those collected from Brownsville or Galveston. As such, we see viral infection alters the microbiome of mosquitoes in a host-line dependant manner in both lab-reared and field-collected mosquitoes

To delve further into the difference seen in the lab-reared lines we examined bacterial taxa that differed between each line either in the unexposed, exposed and infected groups which could account for the observed microbiota shifts. In the RGV-lab line, *Lactobacillus*, *Akkermansia*, and *Turicibacter* were enriched in exposed and infected RGV-lab mosquitoes. In contrast to previous work that found *Citrobacter* increased in abundance after a CHIKV infection in *Aedes albopictus* mosquitoes [8], here we saw this genus was enriched in unexposed mosquitoes in both lab lines. We were also interested in correlating microbes that were differentially abundant within a line in infected compared to exposed individuals as these were microbes that could potentially facilitate or interfere with infection respectively. No differentially abundant bacteria were identified in this comparison in the Galveston-lab line but *Raoultella* and *Enterobacteriaceae* were enriched in the exposed group in the RVG-lab line*. Asaia*, a dominant member of the microbiome, was found enriched in Galveston-lab mosquitoes in exposed and infected mosquitoes compared to unexposed, possibly suggesting that *Asaia* could promote infection in this host. Alternatively, *Asaia* may not be affected by an immune response induced by the virus which suppresses other microbiota. Interestingly, despite the presence of *Asaia* in RGV-lab mosquitoes we did not see this taxon differentially abundant in this line. Potentially, this may be due to its overwhelming dominance of the microbiome in the RGV-lab line which meant that it was challenging to see distinctions after infection or exposure, or possibly due to differences in the host. While *Asaia* has been studied in *Anopheles* mosquitoes and has been shown to influence *Plasmodium* infections [48, 49], less is known regarding its interaction with arboviruses. Supplementation of *Asaia* species to *Culex pipiens* has been reported to enhance WNV titre in legs and saliva [50]. Conversely, infection of *Culex quinquefasciatus* and *Ae. aegypti* with WNV and ZIKV respectively, appears to have minimal effect on *Asaia* [25]. Further studies investigating *Asaia*-arbovirus interactions are warranted to obtain a more in-depth understanding of these interactions given that *Asaia* has been proposed for control measures. Overall, we see different bacterial taxa modulated in each line further demonstrating that distinct host-microbiome combination respond differently to ZIKV infection.

In contrast to the results from lab-reared mosquitoes, we saw no differentially abundant bacteria between exposed and infected groups in field-collected mosquitoes. This could be related to these mosquitoes harbouring a considerably more diverse microbiome compared to lab-reared mosquitoes. Life histories and the age of field-collected mosquitoes were unknown, but this cohort is likely less uniform than the lab-reared mosquitoes and these factors could further contribute to experimental variation and mask any microbiome signal mediated by ZIKV infection compared to exposed. Alternatively, a lack of differentially abundant bacteria could be due to the microbiome equilibrating post viral exposure. In our experiments we assessed both ZIKV infection and the microbiome at 10 days post exposure to an infectious blood meal. However, the microbiome is dynamic and changes over the course of the mosquito’s life, and these changes may conceal initial differences that influenced virus infection at the time of blood feeding [51]. Supporting this is the finding that alterations in the microbiome were less pronounced in ZIKV-infected mosquitoes at 21 compared to seven dpi [25], suggesting that microbiomes reverted toward the non-infectious state over time, potentially as the immune response returns to baseline or due to prolonged sugar feeding which subsequently alters microbiome composition [51].

It is well established that pathogen infection or microbiome colonization elicits an immune response in the mosquito and, in turn, these immune pathways interfere and control gut-associated bacteria and arboviruses, respectively [26, 27]. To that end, it has been postulated that insect immune pathways evolved alongside microbes and are used to maintain homeostasis of the gut microbiome. These processes are particularly important for mosquitoes as they are immersed within these microbes in the larval environment [52]. Therefore, differences in immune profiles, microbiome compositions, and susceptibility of microbes to host pathways could potentially explain the differential responses of the microbiomes of distinct mosquito lines to viral infection. Supporting this is the finding that distinct global transcription profiles are observed in different host backgrounds in response to viral infection or microbial colonization [53–57]. Therefore, variable host responses to infection could mediate divergent microbiota compositions post viral infection. Further comparative studies examining the variation in the transcriptional response to infection in a controlled system, investigating how host pathways influence microbiota, would likely provide insights to the mechanisms mediating variability seen in our studies.

In our study we employed an approach that exploited the natural variation in the microbiome in mosquitoes and correlated this to viral infection outcomes. A benefit of this design to investigate host-microbe-pathogen interactions is the lack of artificial perturbation of the microbiome, which can have adverse effects on the host. However, we do appreciate there are several caveats to our experiments which should be considered when interpreting our results. Unfortunately, the microbiome analysis did not include negative controls, as our sequencing was performed before the microbiome field fully appreciated the importance of such controls. We did however test for potential kit-derived contaminants by applying a previously described method [3] to our dataset. However, this resulted in genuine mosquito symbionts such as *Asaia* and *Serratia* flagged as contaminants, which are well known components of the mosquito microbiome. We therefore did not subtract these taxa from our analysis but provide a table showing the relative abundances of all OTUs in the dataset, highlighting the high and low abundance taxa as supplementary data (Table S2). Known gut-associated bacteria of mosquitoes that are in high abundance are unlikely to have infiltrated our dataset as contaminants. This is supported by Fierer et al. [58], which posits that contamination is mostly an issue in low biomass samples. We quantified the microbiome load using the commonly used 2^-ΔΔCt^ method (16S/S7) [34, 59–61], and observed a comparable ratio of bacterial to host genomes, indicating there was a considerable bacterial loads in these samples, suggesting the effects from contaminants from reagents would likely be negligible. However, without the specific negative controls for the amplicon sequencing we cannot unambiguously conclude this with certainty so caution should be applied when interpreting these findings. Secondly, while field caught mosquitoes are desirable to use in experiments given their lab-reared counterparts have dramatically different microbiomes, they do impose other challenges such as the unknown variables regarding their genetics, age, life history, exposure to pathogens, and previous blood feeding status. Procedures which transplant field microbiomes to mosquitoes in the lab [62–64] could be used in conjunction with approaches here to overcome some of these limitations. Despite these caveats and challenges, our approach did illuminate our understanding of mosquito-microbiome-pathogen interactions.

In conclusion, we show that exposure to, or infection with, ZIKV in *Ae. aegypti* lines alters their microbiome in distinct fashions dependant on the mosquito line. These differences were observed in both lab-reared and field-collected mosquitoes. Different bacterial taxa were modulated between mosquito lines which may be due to bacterial alteration of viral infection or the susceptibility of bacterial taxa after virus infection, which is likely mediated by host pathways. Our results highlight how variation of the microbiomes of mosquitoes needs to be considered for interpretation of lab-based experiments and implementation of microbial-based strategies for vector-borne disease.

## Electronic supplementary material

Below is the link to the electronic supplementary material.

Supplementary Material 1

Supplementary Material 2

Supplementary Material 3

## Data Availability

The datasets generated, analysed, and supporting the conclusions of this article are available at **PRJNA1113645**, the detailed per-sample accession numbers are in Table S1. The R code used to analyse the data and produce all figures is publicly available at [https://github.com/grant-hughes-lab/Zika-microbiome-interactions] (https://url.uk.m.mimecastprotect.com/s/nfngCE0EpSpk69oiNfZT7Do4j?domain=github.com) under zenodo id [https://doi.org/10.5281/zenodo.14786744] (https://doi.org/10.5281/zenodo.14786744).

## References

[1] Guégan M, et al. The mosquito holobiont: fresh insight into mosquito-microbiota interactions. Microbiome. 2018;6:1–17. 29554951 10.1186/s40168-018-0435-2 PMC5859429

[2] Cansado-Utrilla C, et al. The microbiome and mosquito vectorial capacity: rich potential for discovery and translation. Microbiome. 2021;9(1):111. 34006334 10.1186/s40168-021-01073-2 PMC8132434

[3] Seabourn PS, et al. Aedes albopictus microbiome derives from environmental sources and partitions across distinct host tissues. Microbiologyopen. 2023;12(3):e 1364. 10.1002/mbo3.1364 PMC10261752 37379424

[4] Hughes GL, et al. Native microbiome impedes vertical transmission of Wolbachia in anopheles mosquitoes. Proc Natl Acad Sci, India, Sect B Biol Sci. 2014;111(34):12498–503. 10.1073/pnas.1408888111 PMC4151774 25114252

[5] Hegde S, et al. Interkingdom interactions shape the fungal microbiome of mosquitoes. Anim Microbiome. 2024;6(1):11. 38454530 10.1186/s42523-024-00298-4 PMC10921588

[6] Kozlova EV, et al. Microbial interactions in the mosquito gut determine Serratia colonization and blood-feeding propensity. The ISME J. 2021;15(1):93–108. 32895494 10.1038/s41396-020-00763-3 PMC7852612

[7] Brettell LE, et al. Mosquitoes reared in nearby insectaries at the same institution have significantly divergent microbiomes. Environ Microbiol. 2025;27(1):e70027. 39779320 10.1111/1462-2920.70027 PMC11711076

[8] Zouache K, et al. Chikungunya virus impacts the diversity of symbiotic bacteria in mosquito vector. Mol Ecol. 2012;21(9):2297–309. 22433115 10.1111/j.1365-294X.2012.05526.x

[9] Arévalo-Cortés A, et al. Association of midgut bacteria and their metabolic pathways with Zika infection and insecticide resistance in Colombian Aedes aegypti populations. Viruses. 2022;14(10):2197. 36298752 10.3390/v14102197 PMC9609292

[10] Muturi EJ, et al. Midgut fungal and bacterial microbiota of Aedes triseriatus and Aedes japonicus shift in response to La Crosse virus infection. Mol Ecol. 2016;25(16):4075–90. 27357374 10.1111/mec.13741

[11] Tesh RB, Gubler DJ, Rosen L. Variation among goegraphic strains of Aedes albopictus in susceptibility to infection with chikungunya virus. The Am J Trop Med And Hyg. 1976;25(2):326–35. 1259092 10.4269/ajtmh.1976.25.326

[12] Bennett KE, et al. Variation in vector competence for dengue 2 virus among 24 collections of Aedes aegypti from Mexico and the United States. The Am J Trop Med And Hyg. 2002;67(1):85–92. 12363070 10.4269/ajtmh.2002.67.85

[13] Roundy CM, et al. Variation in Aedes aegypti mosquito competence for Zika virus transmission. Emerg. Infect. Dis. 2017;23(4):625. 28287375 10.3201/eid2304.161484 PMC5367433

[14] Kilpatrick AM, et al. Spatial and temporal variation in vector competence of Culex pipiens and cx. restuans mosquitoes for West Nile virus. The Am J Trop Med And Hyg. 2010;83(3):607. 20810828 10.4269/ajtmh.2010.10-0005 PMC2929059

[15] Gubler DJ, Rosen L. Variation among geographic strains of Aedes albopictus in susceptibility to infection with dengue viruses. The Am J Trop Med And Hyg. 1976;25(2):318–25. 1259091 10.4269/ajtmh.1976.25.318

[16] Ramirez JL, et al. Chromobacterium Csp_P reduces malaria and dengue infection in vector mosquitoes and has entomopathogenic and in vitro anti-pathogen activities. PLoS Pathog. 2014;10(10):e1004398. 25340821 10.1371/journal.ppat.1004398 PMC4207801

[17] Ramirez JL, et al. Reciprocal tripartite interactions between the Aedes aegypti midgut microbiota, innate immune system and dengue virus influences vector competence. PLoS Negl Trop Dis. 2012;6(3):e 1561. 10.1371/journal.pntd.0001561 PMC3295821 22413032

[18] Apte-Deshpande AD, et al. Serratia odorifera mediated enhancement in susceptibility of Aedes aegypti for chikungunya virus. Indian J Med Res. 2014;139(5):762–68. 25027087 PMC4140042

[19] Dickson LB, et al. Carryover effects of larval exposure to different environmental bacteria drive adult trait variation in a mosquito vector. Sci Adv. 2017;3(8):e1700585. 28835919 10.1126/sciadv.1700585 PMC5559213

[20] Accoti A, et al. The influence of the larval microbiome on susceptibility to Zika virus is mosquito genotype-dependent. PLoS Pathog. 2023;19(10):e1011727. 37903174 10.1371/journal.ppat.1011727 PMC10635568

[21] Louie W, Coffey LL. Microbial composition in larval water enhances Aedes aegypti development but reduces transmissibility of Zika virus. Msphere. 2021;6(6):e00687–21. 34878293 10.1128/msphere.00687-21 PMC8653847

[22] Becker MV, et al. Reduced microbe abundance in an urban larval development container increases Aedes aegypti susceptibility to Zika virus. PLoS Pathog. 2025;21(5):e1013154. 40388498 10.1371/journal.ppat.1013154 PMC12121923

[23] Garrigós M, et al. Interactions between West Nile virus and the microbiota of Culex pipiens vectors: a literature review. Pathogens. 2023;12(11):1287. 38003752 10.3390/pathogens12111287 PMC10675824

[24] Villegas LE, et al. Zika virus infection modulates the bacterial diversity associated with Aedes aegypti as revealed by metagenomic analysis. PLoS One. 2018;13(1):e0190352. 29293631 10.1371/journal.pone.0190352 PMC5749803

[25] Shi C, et al. Bidirectional interactions between arboviruses and the bacterial and viral microbiota in Aedes aegypti and Culex quinquefasciatus. MBio. 2022;13(5):e01021–22. 36069449 10.1128/mbio.01021-22 PMC9600335

[26] Gabrieli P, et al. Mosquito trilogy: microbiota, immunity and pathogens, and their implications for the control of disease transmission. Front Microbiol. 2021;12:630438. 33889137 10.3389/fmicb.2021.630438 PMC8056039

[27] Cai JA, Christophides GK. Immune interactions between mosquitoes and microbes during midgut colonization. Curr Opin Insect Sci. 2024;101195. 10.1016/j.cois.2024.101195 38552792

[28] Wu P, et al. A gut commensal bacterium promotes mosquito permissiveness to arboviruses. Cell Host Microbe. 2019;25(1):101–12. e5. 30595552 10.1016/j.chom.2018.11.004

[29] Saraiva RG, et al. Chromobacterium spp. mediate their anti-plasmodium activity through secretion of the histone deacetylase inhibitor romidepsin. Sci Rep. 2018;8(1):6176. 29670144 10.1038/s41598-018-24296-0 PMC5906607

[30] Zhang L, et al. A naturally isolated symbiotic bacterium suppresses flavivirus transmission by Aedes mosquitoes. Science. 2024;384(6693):eadn 9524. 10.1126/science.adn9524 38669573

[31] Chabanol E, et al. Antibiotic treatment in anopheles coluzzii affects carbon and nitrogen metabolism. Pathogens. 2020;9(9):679. 32825534 10.3390/pathogens9090679 PMC7558193

[32] Ballard J, Melvin R. Tetracycline treatment influences mitochondrial metabolism and mtDNA density two generations after treatment in Drosophila. Insect Mol Biol. 2007;16(6):799–802. 18093008 10.1111/j.1365-2583.2007.00760.x

[33] Guerbois M, et al. Outbreak of Zika virus infection, Chiapas State, Mexico, 2015, and first confirmed transmission by Aedes aegypti mosquitoes in the Americas. The J Infect Dis. 2016;214(9):1349–56. 27436433 10.1093/infdis/jiw302 PMC5079363

[34] Hegde S, et al. Microbiome interaction networks and community structure from laboratory-reared and field-collected Aedes aegypti, Aedes albopictus, and Culex quinquefasciatus mosquito vectors. Front Microbiol. 2018;9:405381. 10.3389/fmicb.2018.02160 PMC6140713 30250462

[35] Weisburg WG, et al. 16S ribosomal DNA amplification for phylogenetic study. J Bacteriol. 1991;173(2):697–703. 1987160 10.1128/jb.173.2.697-703.1991 PMC207061

[36] Isoe J, et al. Defects in coatomer protein I (COPI) transport cause blood feeding-induced mortality in yellow fever mosquitoes. Proc Natl Acad Sci, India, Sect B Biol Sci. 2011;108(24):E211–17. 10.1073/pnas.1102637108 PMC3116422 21628559

[37] Livak KJ, Schmittgen TD. Analysis of relative gene expression data using real-time quantitative PCR and the 2− ΔΔCT method. Methods. 2001;25(4):402–08. 11846609 10.1006/meth.2001.1262

[38] Kassambara A. Ggpubr: ‘ggplot2‘ based publication ready plots. R Package Version. 2018;p.2.

[39] R Core Team. R: a language and environment for statistical computing. 2023.

[40] Klindworth A, et al. Evaluation of general 16S ribosomal RNA gene PCR primers for classical and next-generation sequencing-based diversity studies. Nucleic Acids Res. 2013;41(1):e1–1. 22933715 10.1093/nar/gks808 PMC3592464

[41] Quast C, et al. The SILVA ribosomal RNA gene database project: improved data processing and web-based tools. Nucleic Acids Res. 2012;41(D1):D590–96. 23193283 10.1093/nar/gks1219 PMC3531112

[42] McMurdie PJ, Holmes S. Phyloseq: an R package for reproducible interactive analysis and graphics of microbiome census data. PLoS One. 2013;8(4):e61217. 23630581 10.1371/journal.pone.0061217 PMC3632530

[43] Oksanen JSG, Blanchet F, Kindt R, Legendre P, Minchin P, O’Hara R, Solymos P, Stevens M, Szoecs E, Wagner H, Barbour M, Bedward M, Bolker B, Borcard D, Carvalho G, Chirico M, De Caceres M, Durand S, Evangelista H, FitzJohn R, Friendly M. Vegan: Community Ecology Package. 2022.

[44] Arbizu PM. pairwiseAdonis: Pairwise Multilevel Comparison using Adonis. 2017.

[45] Wickham H. ggplot2. Wiley Interdiscip Rev Comput Stat. 2011;3(2):180–85.

[46] Lin H, Peddada SD. Analysis of compositions of microbiomes with bias correction. Nat Commun. 2020;11(1):3514. 32665548 10.1038/s41467-020-17041-7 PMC7360769

[47] Kolde R, Kolde MR. Package ‘pheatmap’. R Package. 2015;1(7):790.

[48] Tatsinkou Maffo CG, et al. Contrasting patterns of Asaia association with plasmodium falciparum between field-collected anopheles gambiae and anopheles coluzzii from Cameroon. Microbiol Spectr. 2024;12(12):e00567–24. 39530680 10.1128/spectrum.00567-24 PMC11619320

[49] Bassene H, et al. 16S metagenomic comparison of plasmodium falciparum-infected and noninfected anopheles gambiae and anopheles funestus microbiota from Senegal. The Am J Trop Med And Hyg. 2018;99(6):1489. 30350766 10.4269/ajtmh.18-0263 PMC6283497

[50] Roman A, et al. Asaia spp. exposure for improving mosquito mass-rearing, and the effects on Culex pipiens pipiens vector competence for West Nile virus. PLoS One. 2025;20(8):e0330703. 40839618 10.1371/journal.pone.0330703 PMC12370026

[51] Wang Y, et al. Dynamic gut microbiome across life history of the malaria mosquito anopheles gambiae in Kenya. PLoS One. 2011;6(9):e24767. 21957459 10.1371/journal.pone.0024767 PMC3177825

[52] Hanson MA. When the microbiome shapes the host: immune evolution implications for infectious disease. Phil Trans Of The R Soc B. 2024;379(1901):20230061. 10.1098/rstb.2023.0061 PMC10945400 38497259

[53] Etebari K, et al. Global transcriptome analysis of Aedes aegypti mosquitoes in response to Zika virus infection. MSphere. 2017;2(6). 10.1128/msphere.00456-17. 10.1128/mSphere.00456-17 PMC5700376 29202041

[54] Hyde J, et al. Limited influence of the microbiome on the transcriptional profile of female Aedes aegypti mosquitoes. Sci Rep. 2020;10(1):10880. 32616765 10.1038/s41598-020-67811-y PMC7331810

[55] Jia N, et al. Transcriptome analysis of response to Zika virus infection in two aedes albopictus strains with different vector competence. Int J Mol Sci. 2023;24(5):4257. 36901688 10.3390/ijms24054257 PMC10002152

[56] Vogel KJ, et al. Transcriptome sequencing reveals large-scale changes in axenic Aedes aegypti larvae. PLoS Negl Trop Dis. 2017;11(1):e0005273. 28060822 10.1371/journal.pntd.0005273 PMC5245907

[57] Wang S, et al. A cell atlas of the adult female Aedes aegypti midgut revealed by single-cell RNA sequencing. Sci Data. 2024;11(1):587. 38839790 10.1038/s41597-024-03432-8 PMC11153528

[58] Fierer N, et al. Guidelines for preventing and reporting contamination in low-biomass microbiome studies. Nat Microbiol. 2025:p.1–11. 10.1038/s41564-025-02035-2 40542287

[59] Habtewold T, Duchateau L, Christophides GK. Flow cytometry analysis of the microbiota associated with the midguts of vector mosquitoes. Parasit Vectors. 2016;9(1):167. 27004717 10.1186/s13071-016-1438-0 PMC4802834

[60] MacLeod HJ, Dimopoulos G, Short SM. Larval diet abundance influences size and composition of the midgut microbiota of Aedes aegypti mosquitoes. Front Microbiol. 2021;12:645362. 34220739 10.3389/fmicb.2021.645362 PMC8249813

[61] Wang X, et al. Bacterial microbiota assemblage in Aedes albopictus mosquitoes and its impacts on larval development. Mol Ecol. 2018;27(14):2972–85. 29845688 10.1111/mec.14732 PMC6380897

[62] Coon KL, Hegde S, Hughes GL. Interspecies microbiome transplantation recapitulates microbial acquisition in mosquitoes. Microbiome. 2022;10(1):58. 35410630 10.1186/s40168-022-01256-5 PMC8996512

[63] Zhao SY, Hughes GL, Coon KL. A cryopreservation method to recover laboratory-and field-derived bacterial communities from mosquito larval habitats. PLoS Negl Trop Dis. 2023;17(4):e0011234. 37018374 10.1371/journal.pntd.0011234 PMC10109488

[64] LaReau JC, et al. Introducing an environmental microbiome to axenic Aedes aegypti mosquitoes documents bacterial responses to a blood meal. Appl Environ Microb. 2023;89(12):e00959–23. 10.1128/aem.00959-23 PMC10734439 38014951

